# In-Match Physical Requirements and Team Performance in Cerebral Palsy Football Across a Competitive Season

**DOI:** 10.3390/s25103193

**Published:** 2025-05-19

**Authors:** Juan Francisco Maggiolo, Alejandro Javaloyes, Manuel Moya-Ramón, Iván Peña-González

**Affiliations:** 1 Sports Research Centre, Department of Sport Sciences, Miguel Hernández University of Elche, 03202 Alicante, Spain; lic.franciscomaggiolo@gmail.com (J.F.M.); ajavaloyes@umh.es (A.J.); ipena@umh.es (I.P.-G.); 2 Spanish Federation of Sports for People with Cerebral Palsy and Acquired Brain Injury (FEDPC), 28011 Madrid, Spain

**Keywords:** cerebral palsy, time–motion analysis, physical performance, para-sport, paralympic

## Abstract

**Highlights:**

**What are the main findings?**

- Total distance covered—especially at low intensity—seems to be the strongest predictor of team performance in CP football over an entire national season.
- No significant differences were found in high-intensity actions or ball contacts between higher- and lower-ranked teams.

**What is the implication of the main finding?**

- Physical efficiency and movement control, rather than just explosive efforts, appear to be key contributors to competitive success in CP football.
- Coaches and practitioners should prioritize training strategies that enhance sustained physical output and tactical organization.

**Abstract:**

This study aimed to analyze the in-match physical requirements of cerebral palsy football (CP football) players over an entire national league season (56 matches) and their relationship with team performance-related metrics. Key variables examined included total distance, distance at different intensities, acceleration/deceleration patterns, and ball contacts at various intensities. Statistical analyses (one-way ANOVA, *t*-tests, Pearson’s correlations, and multiple linear regressions) were conducted to identify differences and predictive relationships between these physical requirements and team success indicators (ranking position, points, and goal-related outcomes). Higher-ranked teams covered significantly greater total and walking distances (t = 2.73 and 3.09, *p* < 0.01). Total distance had the strongest relationship with team performance (r = 0.91–0.99, R^2^ = 0.82–0.99), followed by walking and low-intensity distances (r = 0.71–0.92, R^2^ = 0.66–0.88) and certain acceleration/deceleration actions. In contrast, no significant differences were found in high-intensity actions or ball contact patterns between teams with different performance-related outcomes. These findings suggest that success in CP football is closely related to total distance, particularly at low intensities, indicating a strong connection between physical requirements and tactical strategies. These insights are valuable for coaches and sports professionals, helping to optimize match strategies and training approaches to enhance team performance.

## 1. Introduction

Cerebral palsy football (CP football) is a para-sport designed for athletes with neurological impairments that affect motor function, including hypertonia, ataxia, and athetosis. The sport follows a 7-a-side format, with modified rules to accommodate the functional limitations of the players while maintaining the integrity and competitiveness of the game [1]. To ensure fair competition, CP football uses a functional classification system (FT1–FT3), in which players are categorized based on the severity of their impairment [2]. The game is played on a reduced field (70-m × 50-m) with reduced goals (5-m × 2-m), and it is played in two 30 min halves. The main rule adaptation is the removal of the offside rule, which allows for a more continuous and dynamic style of play. Given the neuromuscular challenges faced by CP football players, understanding the physical requirements of the sport is crucial for optimizing training methodologies, tactical approaches, and player performance assessment.

In recent years, research on cerebral palsy (CP) football has expanded exponentially, significantly enhancing current knowledge on the physical demands of competition [3,4,5,6], as well as the tactical and strategic requirements associated with sporting success [7,8,9]. Emerging literature in this field has highlighted the critical role of physical performance—such as sprinting ability, change of direction, and dribbling skills—in determining players’ competitive success [10]. Moreover, players who demonstrate superior physical performance in field-based tests also tend to exhibit higher physical demands during match play [11].

Recent advances in sports science and technology have enabled a more detailed analysis of the physical performance of CP football players through global positioning systems (GPS) and inertial measurement units (IMUs). These wearable tracking devices allow for precise quantification of key performance metrics, including total distance covered, movement intensities, accelerations, and decelerations, among others. Compared to mainstream football, CP football players have been shown to cover less distance at high intensities, perform fewer high-intensity accelerations and decelerations, and exhibit lower peak sprint speeds due to their motor impairments [3,4].

In mainstream football, there is ongoing debate regarding the influence of a team’s competitive level on the players’ in-match physical requirements. Conventionally, greater physical requirements have been associated with athletes competing at higher competitive levels [12,13,14]. However, evidence suggests that these physical requirements may fluctuate depending on contextual variables, including team ranking, match outcomes, and the quality of the opposition [15,16]. To date, only one study in CP football has examined the in-match physical requirements of international teams categorized as top-ranked versus bottom-ranked, indicating that players from top-ranked teams cover greater distances at low intensities, while no significant differences are observed in high-intensity actions compared to their bottom-ranked counterparts [6]. These findings underscore the pivotal role of physical requirements in CP football; nevertheless, the degree to which distinct physical performance metrics distinguish teams based on their competitive success remains largely uninvestigated.

In elite mainstream football, research has shown that top-ranked teams tend to control possession for longer periods, which results in greater distances covered at low intensities and fewer explosive actions, whereas teams with lower rankings often engage in more transitions and high-intensity actions due to their defensive positioning [17,18]. Similar trends may be expected in CP football, but there is limited research investigating how physical requirements vary between teams of different competitive levels. Given the influence of functional classification and the varying severity of impairments among players, understanding the relationship between in-match physical requirements and competitive success in CP football is particularly important for optimizing training strategies, player selection, and game models.

This study aimed to analyze the physical requirements of CP football players across an entire competitive season in the First Division of the FEDPC National 7-a-side Football League (N7FL). Specifically, it seeks to determine whether in-match physical requirement metrics—such as total distance covered, movement intensity distribution, and acceleration/deceleration patterns—are associated with and predictive of team success, measured in terms of ranking position, points obtained, and goal-related outcomes. We hypothesized that all in-match physical performance metrics would be more favorable in higher-ranked teams, with particular emphasis on high-intensity variables—such as high-speed running—being the most discriminative indicators of competitive success. By examining these relationships, this study will provide novel insights into the key performance indicators in CP football, offering practical implications for coaches, sports scientists, and classification specialists working in para-sport contexts. Additionally, the findings could contribute to the ongoing development of training programs tailored to the unique physical demands of CP football, ultimately enhancing player performance and competitive balance at both national and international levels.

## 2. Materials and Methods

### 2.1. Design

This study was conducted over the course of an entire season (2024) in the First Division of the FEDPC N7FL. The physical demands of participating players were monitored in every match, encompassing all 56 games of the competition (with each team playing six matches). This cross-sectional study aimed to analyze the physical demands associated with the most successful teams throughout a full season of CP football.

### 2.2. Participants

All players registered with the FEDPC who participated in at least one match as a starter were included in this study, except for goalkeepers, who were excluded due to having distinct physical demands compared to outfield players. A total of 68 CP football players (FT1: 8; FT2: 51; FT3: 9) from the seven teams competing in the First Division of the FEDPC N7FL met the inclusion criteria and were ultimately included in this study. All participants received detailed information about the study’s objectives and voluntarily provided written informed consent, adhering to the principles of the Declaration of Helsinki. This study received approval from the ethics committee of the researchers’ affiliated university (REF: ADH.DES.IPG.JFM.24).

### 2.3. Procedures

The physical demands of players during competition were assessed using a wearable device (“OLIs”), which recorded data at a sampling frequency of 10 Hz (Oliver IMU^®^, Barcelona, Spain). At the start of each match, OLIs were affixed to all six outfield players on each team. Unlike other comparable devices, the OLI is positioned on the back of the player’s dominant leg, enabling the measurement of both the frequency and velocity of ball contacts. The variables extracted from the OLI device included the following:

• Maximal velocity (km·h^−1^): The highest recorded movement velocity achieved by the player during the match.
• Total distance (m): The total meters covered by the player during the match.
• Walking (m): Distance covered while walking (<6 km·h^−1^).
• LI running (m): Distance covered at low-intensity running (6–12 km·h^−1^).
• MI running (m): Distance covered at moderate-intensity running (12–18 km·h^−1^).
• HI running (m): Distance covered at high-intensity running (>18 km·h^−1^).
• Ball contacts (n): Total number of contacts with the ball.
• LI ball contacts (n): Number of low-intensity (ankle velocity < 11 m·s^−1^ but acceleration > 20 G) contacts with the ball.
• MI ball contacts (n): Number of moderate-intensity (ankle velocity 11–15 m·s^−1^) contacts with the ball.
• HI ball contacts (n): Number of high-intensity (ankle velocity > 15 m·s^−1^) contacts with the ball.
• Striking force (km·h^−1^): Maximum velocity reached during a ball strike in the match.
• MI accelerations (m): Distance covered with moderate-intensity accelerations (>2 to 3 m·s^−2^).
• HI accelerations (m): Distance covered with high-intensity accelerations (>3 m·s^−2^).
• MI decelerations (m): Distance covered with moderate-intensity decelerations (<−2 to −3 m·s^−2^).
• HI decelerations (m): Distance covered with high-intensity decelerations (<−3 m·s^−2^).

All variables, except for maximal velocity and maximum striking force, were adjusted based on each player’s total playing time to derive “per minute” values. This standardization was implemented to minimize potential bias resulting from variations in playing time among players.

Additionally, the following team-performance-related outcomes were collected for each participating team at the conclusion of the competition:

• Ranking Position: Final standing of the team in the league (from 1st to 7th position).
• Points: Total number of points accumulated by the team at the end of the season, awarded as follows: 3 points per win, 1 point per draw, and 0 points per loss.
• Goals For: Total number of goals scored by the team throughout the competition.
• Goals Against: Total number of goals conceded by the team during the competition.
• Goal Difference: The difference between goals scored and goals conceded, calculated as Goal Difference = Goals For − Goals Against.

### 2.4. Statistical Analysis

Physical requirement data were averaged based on the number of matches each player played. Players were categorized according to their team’s final ranking (from 1st to 7th) and further classified based on whether their team secured a playoff position (1st to 4th) or not (5th to 7th). A Shapiro–Wilk test was conducted to assess the normal distribution of each analyzed variable. A one-way analysis of variance (ANOVA) was performed to examine potential differences in physical demands among players from teams with different ranking positions. Effect size was estimated using partial eta-squared (ηp^2^) and interpreted as follows: small (ηp^2^ = 0.01–0.05), moderate (ηp^2^ = 0.06–0.13), and large (ηp^2^ > 0.13) [19]. An independent samples *t*-test was conducted to examine potential differences in in-match physical demands between players from teams that qualified for the playoffs and those from non-playoff teams. Pairwise effect size at 95% CI was calculated as Cohen’s *d* between playoff and non-playoff groups. Cohen’s *d* was interpreted as follows: trivial (*d* < 0.20), small (*d* = 0.20–0.50), moderate (*d* = 0.50–0.80), and large (*d* > 0.80) [19]. Subsequently, the physical requirement values of the players from each team were averaged. Pearson’s correlation coefficients (r) were calculated to assess the relationships between in-match physical requirements and the teams’ performance-related outcomes. Correlation strength was interpreted as trivial (r < 0.09), small (r = 0.10–0.29), moderate (r = 0.30–0.49), high (r = 0.50–0.69), very high (r = 0.70–0.89), and almost perfect (r > 0.90) [20]. For the physical requirement variables that showed significant correlations with any of the performance-related outcomes, multiple linear regression analyses were conducted to determine the extent to which in-match physical requirements predicted the teams’ competitive outcomes. Additionally, a stepwise linear regression analysis was performed for each performance-related outcome to identify the model that best predicted each outcome. All data were processed and analyzed using Microsoft Excel (Microsoft, Seattle, WA, USA) and JASP software (JASP for Windows, version 0.13, Amsterdam, The Netherlands). The level of significance was set at *p* < 0.05.

## 3. Results

Shapiro–Wilk tests showed a normal distribution for each variable (*p* > 0.05). The results of the ANOVA (Table 1) indicated that no statistically significant differences were observed among teams ranked from 1st to 7th place in the league for any of the studied variables. Effect sizes were generally small to moderate (ηp^2^ < 0.14) for most variables. However, large effect sizes were observed for total distance covered (ηp^2^ = 0.15), walking distance (ηp^2^ = 0.17), and striking force (ηp^2^ = 0.17). Additionally, both total distance and walking distance exhibited a tendency to be greater in teams with higher rankings (total distance: 68.44 ± 12.23 m for the 1st place team vs. 50.06 ± 18.16 m for the 7th place team; walking distance: 33.16 ± 5.99 m for the 1st place team vs. 24.68 ± 10.49 m for the 7th place team), although these differences did not reach statistical significance. Similarly, no significant differences were observed for most in-match physical demands between players from playoff-qualifying teams (1st to 4th) and those from non-playoff teams (5th to 7th). However, exceptions were found for total distance (t = 2.73; *p* = 0.01; *d* = 0.67 [0.17; 1.16]), walking distance (t = 3.09; *p* < 0.01; *d* = 0.75 [0.26; 1.25]), and striking force (t = 2.18; *p* = 0.03; *d* = 0.25 [0.04; 1.02]), which differed between groups.

Pearson’s correlation analysis revealed significant associations between several physical requirement variables and the team’s performance-related outcomes (Table 2). Total distance showed strong and significant correlations with all performance-related outcomes (r = 0.91 to 0.99, *p* < 0.001), emerging as the most consistently associated variable with team performance. Additionally, walking distance, low and moderate-intensity running distances, and moderate-intensity accelerations exhibited strong correlations with most performance-related outcomes. High-intensity accelerations (r = 0.79, *p* < 0.05) and high-intensity decelerations (r = 0.76, *p* < 0.05) showed significant positive correlations with goals scored, while moderate-intensity decelerations (r = −0.78, *p* < 0.05) were negatively associated with goals conceded. Maximum velocity also displayed significant relationships with points, goals conceded, and goal difference (r = 0.78 to 0.80, *p* < 0.05), whereas other variables, such as ball contacts, exhibited weaker or non-significant correlations.

Finally, multiple linear regression analyses identified predictive relationships between in-match physical requirement variables and team performance-related outcomes (Table 3). Total distance emerged as the primary predictor in stepwise models for points obtained (R^2^ = 0.95, *p* < 0.001), goals scored (R^2^ = 0.96, *p* < 0.001), goals conceded (R^2^ = 0.95, *p* < 0.001), and goal difference (R^2^ = 0.99, *p* < 0.001), highlighting its strong explanatory power. Other relevant predictors included walking distance (R^2^ = 0.84–0.88, *p* < 0.001) and moderate- (R^2^ = 0.78–0.83, *p* < 0.01) and low-intensity running (R^2^ = 0.66–0.76, *p* < 0.01) for multiple performance factors. Additionally, moderate-intensity accelerations and decelerations demonstrated significant predictive effects, albeit with smaller magnitudes (R^2^ = 0.59–0.80, *p* < 0.05). Maximum velocity also contributed to predicting points, goals conceded, and goal difference (R^2^ = 0.61–0.64, *p* < 0.05), although with a smaller influence compared to total distance covered.

## 4. Discussion

The aim of this study was to examine the in-match physical requirements of CP football players throughout an entire national league season and assess the extent to which they are associated with team performance-related outcome metrics. The main finding of this study was that total distance covered emerged as the most strongly associated variable with team performance, demonstrating significant correlations with all performance-related outcomes. Furthermore, it was identified as the primary predictor in regression models for key competitive metrics, highlighting its substantial explanatory power. Additional relevant predictors included walking distance, low- and moderate-intensity running, and moderate-intensity accelerations, albeit with a lesser impact. These findings underscore the critical role of physical demands in determining competitive success in CP football.

No systematic differences were observed in most in-match physical requirements between players from teams with different ranking positions, except for total distance and walking distance, which significantly differed between players from teams that qualified for the playoffs and those that did not. This finding aligns with previous research in mainstream football, where no systematic differences have been found in most GPS-derived variables, suggesting that performance differences are more closely linked to contextual factors, such as playing position [17], rather than competitive level. Similarly, Henríquez et al. (2023) reported no significant differences in most in-match physical requirements between top-ranked and bottom-ranked international CP football players, except for distances covered at very low or walking intensities (<4 km·h^−1^) [6]. These findings are consistent with those of this study. Higher-ranked CP football players appeared to cover greater total distances during matches, as well as greater distances at low intensity (<6 km·h^−1^). This trend is reflected in the large effect sizes observed in the ANOVA for these variables, despite the absence of statistically significant differences, as well as in the significant differences identified between teams that qualified for the playoffs and those that did not. This suggests that the type of physical requirement most commonly associated with high performance in competition—namely, high-intensity actions—does not appear to be a key differentiating factor between teams of varying competitive levels in CP football [6].

In mainstream football, findings on high-intensity demands remain controversial. Mohr et al. (2003) reported that professional players performed more high-intensity actions during matches than semi-professional players [13], whereas Bradley et al. (2010) demonstrated that the high-intensity activity profile was similar among professional players across different competitive levels, with higher-ranked teams covering greater walking distances [21]. Rampinini et al. (2009) further differentiated between distances covered with and without ball possession, showing that more successful teams covered greater distances while in possession, suggesting a tactical and strategic component influencing physical requirement parameters [16]. A higher level of technical proficiency and tactical organization can be expected in higher-ranked teams, potentially contributing to greater movement efficiency rather than simply covering more distance or performing more high-intensity actions [22]. The increased low-intensity distance covered by higher-ranked CP football players in this study may reflect superior positioning, tactical awareness, and an enhanced ability to control match tempo. This suggests that other factors, such as team tactics, technical ability, or cognitive decision making, may also play crucial roles in determining success. Future research should further explore how physical, technical, and tactical elements interact to influence performance outcomes in CP football, drawing parallels with existing literature in both CP and mainstream football.

The strong correlation and predictive power of total distance covered (r = 0.99, R^2^ = 0.99 for goal difference) and walking distance (r = 0.92, R^2^ = 0.84–0.88) emphasize their importance in CP football. From a tactical perspective, higher-ranked teams may control the game more effectively, maintaining ball possession for longer periods, thereby reducing the need for high-intensity efforts and favoring low-intensity movement patterns [17,18]. This pattern is consistent with findings in mainstream football [23] but could be even more pronounced in CP football due to coordination and muscle power constraints that limit high-intensity actions and favor controlled movements [24]. Tactical organization also plays a key role: teams with well-defined structures optimize collective movement, adjusting positioning with minimal energy expenditure, which could explain the prominence of walking distance [17,22]. Furthermore, greater possession could reduce the frequency of rapid defensive transitions, allowing players to prioritize support play and passing over explosive efforts, as observed in a mainstream football context [13].

The significant correlations found between moderate and high-intensity accelerations and decelerations with goals scored and conceded highlight their relevance in critical game moments [25,26]. In CP football, where accelerations are more demanding due to neuromuscular limitations, these results may reflect peak offensive actions or well-organized defensive efforts to maintain team structure. The lower reliance on high-intensity efforts in top-ranked teams could indicate more efficient high-pressing strategies, with short and compact movements facilitating quick ball recoveries and minimizing physical strain, a critical aspect for players with cerebral palsy [4].

The limited impact of ball contacts in correlation and regression analyses suggests that, in CP football, physical performance may be a stronger predictor of success than technical skills, potentially due to constraints in fine motor coordination and technical execution [27]. These findings are difficult to compare with existing scientific literature, as the number and intensity of ball contacts are not commonly studied variables in football research. The lack of association between the total number of ball contacts, as well as contacts at different intensities, and team performance-related outcomes suggest that teams at different competitive levels do not adopt distinct tactical approaches that would result in a higher number of short passes (as expected in teams favoring positional attacks) or a higher number of long passes (as expected in teams employing more direct attacking styles). In this context, the authors hypothesized that teams with better team performance-related outcomes would have a greater number of ball contacts, as they are presumed to exercise greater control over the game, resulting in more frequent and longer possessions [28]. A higher number of low-intensity ball contacts would also be expected for the same reason. Additionally, it has been demonstrated that, in CP football, goal-scoring actions are often preceded by individual plays, where the goal-scoring player carries the ball—performing low-intensity contacts—before scoring [8].

This study presents several limitations that should be considered when interpreting the findings. The exclusive use of GPS and IMU devices during official competitions, while ensuring ecological validity, restricted the researchers’ ability to collect individual-level data such as anthropometric characteristics or functional motor assessments—variables that have been shown to influence physical performance in CP football. Additionally, contextual match-related factors such as player position, tactical role, scoreline, or opponent level were not accounted for, despite evidence suggesting that such elements modulate physical and technical demands. Although the sample included a relatively large number of players and matches, the findings may not be generalizable beyond the national and international contexts examined, especially considering the variability in player profiles and competitive structures across countries. Future research should aim to address these limitations by incorporating comprehensive player profiling, tactical and positional analysis, and longitudinal designs that explore performance evolution across competitive phases. Expanding the diversity of the sample and examining the impact of playing positions and roles may also enhance the understanding of performance determinants and support the refinement of training and classification strategies in CP football.

### Practical Applications

The findings of this study provide valuable insights for coaches, performance analysts, and sports scientists working with CP football teams. In CP football, total distance covered is the strongest predictor of team success, emphasizing the physical demands of the sport. Higher-ranked teams cover more ground at low intensity, suggesting that match control relies on movement efficiency rather than high-intensity efforts. No significant differences in high-intensity actions were observed between teams of varying competitive levels, indicating that explosive efforts are not a key differentiator in CP football. Instead, low-intensity distances and overall workload appear to be more relevant performance indicators. Accelerations could be decisive in key game moments, highlighting their role in decisive plays, particularly in offensive transitions and defensive recoveries. These findings suggest that CP football performance is more dependent on sustained physical output and tactical organization rather than frequent high-intensity actions or a high volume of technical executions.

## 5. Conclusions

This study demonstrates that success in CP football depends both on the total distance covered and how movement is distributed within a tactical context, possibly adapted to the impact of disability on the game. Among the variables analyzed, total distance emerged as the strongest and most consistent predictor of team performance, highlighting its central role in competitive success. Walking distance and acceleration/deceleration actions also proved relevant, reflecting aspects such as physical efficiency, tactical organization, and effort management. These findings have practical implications for CP football, emphasizing the importance of optimizing controlled movements and physical preparation. Furthermore, they lay the foundation for future research integrating physical metrics with strategic variables to better understand performance in this sport.

## Figures and Tables

**Table 1:** ANOVA results among players from teams ranked 1st to 7th in the league.

Variable	1st (n = 9)	2nd (n = 9)	3rd (n = 10)	4th (n = 10)	5th (n = 9)	6th (n = 11)	7th (n = 10)	ηp^2^
Maximal velocity (km·h^−1^)	22.88 ± 2.33	24.33 ± 2.56	23.46 ± 1.42	23.50 ± 2.53	22.96 ± 2.19	22.55 ± 2.53	22.15 ± 2.59	0.08
Total distance (m)	68.44 ± 12.23	66.38 ± 11.00	65.72 ± 10.83	65.17 ± 18.81	63.68 ± 12.59	54.86 ± 21.58	50.06 ± 18.16	0.15
Walking (m)	33.16 ± 5.99	35.31 ± 4.54	32.35 ± 6.64	31.31 ± 8.03	29.23 ± 5.44	27.87 ± 10.01	24.68 ± 10.49	0.17
LI running (m)	25.88 ± 7.51	21.99 ± 6.22	23.96 ± 4.74	24.26 ± 8.30	24.84 ± 5.87	20.43 ± 9.34	18.53 ± 5.81	0.12
MI running (m)	7.13 ± 2.40	6.53 ± 2.65	7.14 ± 2.19	7.22 ± 3.86	7.16 ± 3.34	4.85 ± 3.40	5.11 ± 2.17	0.11
HI running (m)	0.19 ± 0.19	0.36 ± 0.22	0.21 ± 0.21	0.24 ± 0.23	0.32 ± 0.43	0.18 ± 0.26	0.16 ± 0.18	0.08
Ball contacts (n)	0.29 ± 0.19	0.36 ± 0.12	0.31 ± 0.14	0.21 ± 0.11	0.29 ± 0.16	0.24 ± 0.17	0.27 ± 0.35	0.05
LI ball contacts (n)	0.08 ± 0.05	0.08 ± 0.07	0.07 ± 0.04	0.04 ± 0.02	0.06 ± 0.04	0.05 ± 0.04	0.10 ± 0.18	0.05
MI ball contacts (n)	0.12 ± 0.08	0.11 ± 0.05	0.11 ± 0.05	0.08 ± 0.05	0.13 ± 0.08	0.10 ± 0.07	0.07 ± 0.10	0.08
HI ball contacts (n)	0.09 ± 0.07	0.16 ± 0.09	0.12 ± 0.08	0.09 ± 0.07	0.09 ± 0.05	0.09 ± 0.07	0.10 ± 0.10	0.11
Striking force (km·h^−1^)	46.03 ± 8.08	55.65 ± 14.59	51.19 ± 9.94	46.01 ± 8.95	44.39 ± 4.24	42.63 ± 9.92	46.72 ± 7.46	0.17
MI accelerations (m)	3.50 ± 1.39	3.55 ± 1.16	3.29 ± 0.99	3.49 ± 1.28	3.69 ± 1.90	2.91 ± 1.34	2.82 ± 1.45	0.05
HI accelerations (m)	1.33 ± 0.81	1.41 ± 0.74	1.44 ± 0.66	1.19 ± 0.56	1.53 ± 0.87	1.11 ± 0.59	1.03 ± 0.58	0.07
MI decelerations (m)	3.86 ± 1.43	3.53 ± 1.14	3.85 ± 1.37	3.87 ± 1.43	4.22 ± 1.39	3.20 ± 1.44	3.16 ± 1.91	0.06
HI decelerations (m)	2.04 ± 1.28	1.70 ± 0.62	1.77 ± 0.87	1.72 ± 1.29	2.07 ± 1.33	1.54 ± 0.89	1.43 ± 0.99	0.04

LI: low-intensity; MI: moderate-intensity; HI: high-intensity.

**Table 2:** Pearson’s correlation analysis.

Variable	Ranking Position	Points	Goals For	Goals Against	Goal Difference
Maximal velocity (km·h^−1^)	−0.68	0.78 *	0.72	−0.80 *	0.78 *
Total distance (m)	−0.91 **	0.97 ***	0.98 ***	−0.98 ***	0.99 ***
Walking (m)	−0.94 **	0.91 **	0.92 **	−0.89 **	0.92 **
LI running (m)	−0.71	0.81 *	0.85 *	−0.87 *	0.87 *
MI running (m)	−0.70	0.91 **	0.89 **	−0.90 **	0.91 **
HI running (m)	−0.35	0.54	0.49	−0.59	0.56
Ball contacts (n)	−0.51	0.46	0.49	−0.28	0.37
LI ball contacts (n)	−0.04	−0.10	−0.09	0.42	−0.30
MI ball contacts (n)	−0.54	0.52	0.65	−0.58	0.61
HI ball contacts (n)	−0.41	0.40	0.35	−0.28	0.31
Striking force (km·h^−1^)	−0.54	0.56	0.50	−0.3	0.44
MI accelerations (m)	−0.67	0.86	0.84 *	−0.89 **	0.89 **
HI accelerations (m)	−0.59	0.74	0.79 *	−0.75	0.78 *
MI decelerations (m)	−0.47	0.72	0.72	−0.78 *	0.77 *
HI decelerations (m)	−0.60	0.69	0.76*	−0.71	0.74

LI: low-intensity; MI: moderate-intensity; HI: high-intensity; * *p* < 0.05, ** *p* < 0.01, *** *p* < 0.001.

**Table 3:** Multiple linear regression analyses.

Dependent Variable	Predictor	B	SEB	β	F	R2
Ranking Position	Total Distance (m)	−0.29	0.06	−0.91	22.50 **	0.82
	Walking (m)	−0.57	0.09	−0.94	36.33 ***	0.88 #
Points	Maximal velocity (km·h^−1^)	5.85	2.07	0.78	7.99 *	0.62
	Total Distance (m)	0.76	0.08	0.97	86.73 ***	0.95 #
	Walking (m)	1.37	0.27	0.91	25.24 ***	0.84
	LI running (m)	1.65	0.53	0.81	9.74 *	0.66
	MI running (m)	4.72	0.96	0.91	24.13 ***	0.83
Goals For	Total Distance (m)	1.27	0.11	0.98	132.02 ***	0.96 #
	Walking (m)	2.27	0.45	0.92	25.74 ***	0.84
	LI running (m)	2.87	0.79	0.85	13.17 *	0.73
	MI running (m)	7.59	1.79	0.89	17.98 **	0.78
	MI accelerations (m)	22.20	6.50	0.84	11.68 *	0.70
	HI accelerations (m)	37.57	13.24	0.79	8.06 *	0.62
	HI decelerations (m)	28.26	10.97	0.76	6.64	0.57
Goals Against	Maximal velocity (km·h^−1^)	−16.22	5.46	−0.80	8.81 *	0.64
	Total Distance (m)	−2.08	0.21	−0.98	101.84 ***	0.95 #
	Walking (m)	−3.63	0.83	−0.89	19.10 **	0.79
	LI running (m)	−4.76	1.26	−0.86	14.35 **	0.74
	MI running (m)	−12.62	2.81	−0.90	20.24 **	0.80
	MI accelerations (m)	−38.87	8.79	−0.89	19.55 **	0.80
	MI decelerations (m)	−28.95	10.55	−0.78	7.53 *	0.60
Goal Difference	Maximal velocity (km·h^−1^)	25.07	8.96	0.78	7.82 *	0.61
	Total Distance (m)	3.35	0.14	0.99	563.60 ***	0.99 #
	Walking (m)	5.89	1.16	0.92	25.90 **	0.84
	LI running (m)	7.63	1.91	0.87	15.96 *	0.76
	MI running (m)	20.22	4.20	0.91	23.22 **	0.82
	HI accelerations (m)	96.66	34.87	0.78	7.69 *	0.61
	MI accelerations (m)	61.07	14.24	0.89	18.40 **	0.79
	MI decelerations (m)	45.42	16.87	0.77	7.24 *	0.59

LI: low-intensity; MI: moderate-intensity; HI: high-intensity; * *p* < 0.05, ** *p* < 0.01, *** *p* < 0.001; # main prediction model in the stepwise linear regression analysis.

## Data Availability

The datasets generated and analyzed during this study are not publicly available but may be made available by the corresponding author upon reasonable request. Data will be accessible for a period of three years following the publication of this article. Interested researchers must contact the corresponding author via email with a justified explanation of the intended use of the data. Access will be granted at the discretion of the corresponding author, provided that the request aligns with ethical standards and data protection regulations.
